# Ultrasound-Assisted Hydroxyapatite-Decorated Breath-Figure Polymer-Derived Ceramic Coatings for Ti6Al4V Substrates

**DOI:** 10.1021/acsami.0c08849

**Published:** 2020-10-27

**Authors:** Simone Murchio, Yifu Ding, Giorgio Speranza, Gian Domenico Sorarù, Devid Maniglio

**Affiliations:** † Department of Industrial Engineering, University of Trento, Via Sommarive 9, Povo, 38123 Trento, Italy; ‡ BIOtech, Center for Biomedical Technologies, University of Trento, Via delle Regole 101, 38123 Trento, Italy; ∥ Department of Mechanical Engineering, University of Colorado, 427 UCB, Boulder, Colorado 80309-0427, United States; ⊥ Fondazione Bruno Kessler, Via Sommarive 18, Povo, 38123 Trento, Italy; # Institute of Photonics and Nanotechnologies—CNR, Via alla Cascata 56/C Povo, 38123 Trento, Italy

**Keywords:** breath figure, self-assembly, nanoparticle decoration, ultrasonic atomizer, hydroxyapatite nanoparticles, Pickering emulsion, polymer-derived ceramic, ceramic coating

## Abstract

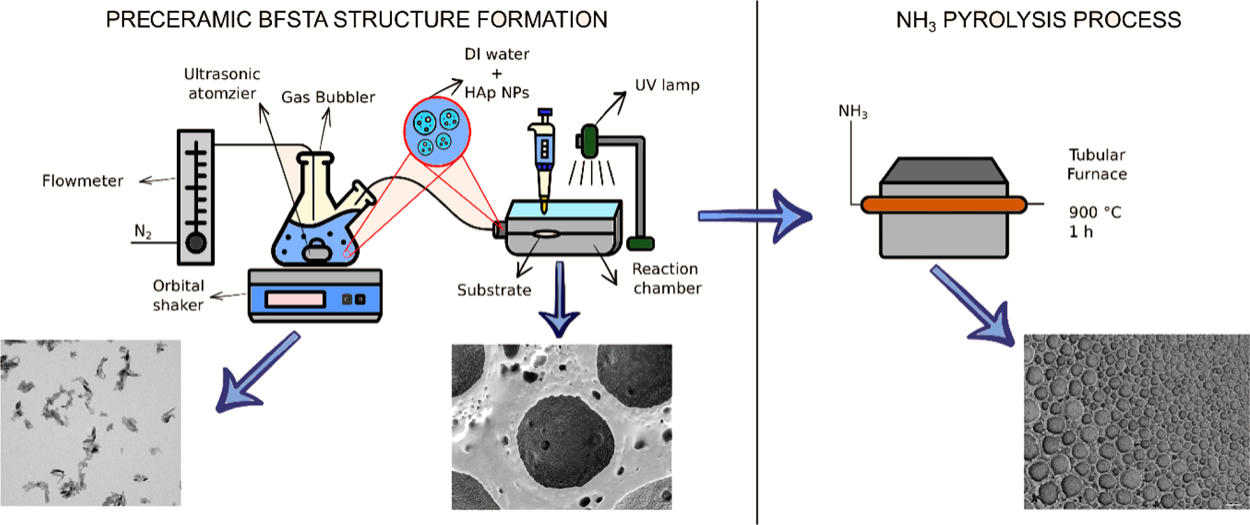

The introduction of nanoparticles (NPs) into the breath-figure-templated self-assembly (BFTSA) process is an increasingly common method to selectively decorate a surface porous structure. In the field of prosthetic devices, besides controlling the morphology and roughness of the structure, NPs can enhance the osteointegration mechanism because of their specific ion release. Among the most widely used NPs, there are silica and hydroxyapatite (HAp). In this work, we propose a novel one-stage method to fabricate NP-decorated surface porous structures that are suitable for prosthetic coating applications. This technique combines the classical direct BFTSA process with the cavitation effect induced by an ultrasonic atomizer that generates a mist of water droplets with embedded NPs. Coatings were successfully obtained by combining a UV cross-linkable polymer precursor, alkoxy silicone, with synthesized HAp NPs, on Ti6Al4V alloy discs. The cross-linked polymeric surface porous structures at selected concentrations were then pyrolyzed in an ammonia atmosphere to obtain a silicon oxynitride (SiON) ceramic coating. Herein, we report the chemical and morphological analyses of both the polymeric and ceramic coatings as well as the effect of NPs at the interface.

## 1 Introduction

Self-assembly techniques can be defined as bottom-up strategies to produce patterned porous structures, suitable for applications at the nanoscale and microscale.^1^ Among these techniques, the breath-figure (BF) process has come to the attention of the scientific community only over the past 2 decades,^2^ even though the mechanism had already been studied and theorized by Lord Rayleigh in 1911.^3^ The breath-figure-templated self-assembly (BFTSA) process is a low-cost, one-stage technique to achieve regular porous patterned structures.^1,2^ It is based on the sinking and evaporation of water droplet arrays from the surface of a polymeric solution. Droplets are generated by moisture condensation, which is induced by the evaporation of a volatile solvent over a cold substrate under a humid environment.^4−6^

The produced patterns, which are highly organized honeycomb structures, can be exploited for a wide range of potential applications, for instance, as membranes,^7^ sensors,^8^ and optoelectronic devices^9^ and also in the biomedical field.^10^ Within the tissue engineering and prosthesis fields, it is well recognized that porosity and roughness control are two key factors to promote cell growth, adhesion, and proliferation.^11−15^ Surface roughness in the range of 1–10 μm^16^ is indeed beneficial for the synthesis, recruitment, and formation of an adsorbed layer of proteins (i.e., integrins), which enhances the cell metabolism and growth.^12^ On the other hand, surface pores, or surface patterns, in the range of 40–250 μm^17^ are effective in affecting the contact guidance of cells.^18^ As a matter of fact, in the cascade of events that leads to osseointegration, osteoblast migration and cell colony formation are more prominent in the presence of 2D–3D patterns, such as pits or pores, than flat surfaces.

The BFTSA structures can be obtained with a wide range of polymeric solutions. Particularly, silicon-based polymers have gained increasing interest as coatings for bulk prosthetic devices because of the possibilities to add elements into the Si network (i.e., nitrogen or carbon) which can enhance the osteoinductive and osteoconductive properties as well as the mechanochemical properties.^19^ Indeed, silicon-based polymers can be used as precursors to produce, via the polymer-derived ceramic (PDC) process,^19^ inorganic coatings for prosthetic devices, as also demonstrated by Carlomagno *et al.*(20,21)

The authors performed pyrolysis of the porous polymer structures under different atmospheres: pure air, nitrogen, and ammonia. Accordingly, different PDC materials with controlled surface pore structures were obtained depending on the pyrolysis atmosphere:^20^ silicon dioxide, SiO_2_ (air atmosphere); silicon oxycarbide, SiOC (N_2_ atmosphere); and silicon oxynitride, SiON (NH_3_ atmosphere). The SiON BFTSA was further evaluated for bioapplications.^21^ Silicon-ion release from the SiON materials was shown to be beneficial to promote osteoblast activation,^22^ a fundamental stage of osseointegration that occurs at the prosthetic–bone interface.^15^ Furthermore, SiON coatings show a thermal expansion coefficient that matches the coefficient of medical-graded titanium alloys (i.e., Ti6Al4V), thus reducing excessive stresses and failure at the interface with the solid titanium surface.^23^

It is worth mentioning that surface coatings can be formed via vapor-phase processes such as plasma spraying, hot isostatic pressing, and thermal spraying^26,27^ or via imbibition-based processes such as dip coating^28^ and Langmuir–Blodgett deposition.^29^ In comparison, the BFTSA process offers additional benefits such as selectively decorating the pore structures by inorganic nanoparticles (NPs).^24,25^ In recent years, this procedure has gained more interest because of either the benefits of forming hybrid polymeric–inorganic patterned films^25^ or the Pickering emulsion phenomenon. Pickering emulsion is another self-assembly phenomenon that takes place at the liquid–liquid interface of a water/oil or oil/water emulsion.^30^ The combination of the Pickering emulsion effect with the BFTSA technique leads to an NP adsorption at the air–water–polymer three-phase contact that mechanically hinders the droplet coalescence. This effect, in turn, increases both the pore regularity and circularity.^24^ Examples of the reported inorganic particles are silica,^31^ gold,^32^ TiO_2_,^33^ and hydroxyapatite (HAp) NPs.^34^ For biomedical applications, HAp is highly desirable because it releases calcium and phosphate ions *in situ*.^35,36^ These ions, combined with Si^4+^, can enhance the osseointegration, thus guaranteeing a better mechanical interlocking between the prosthetic device and the surrounding bone tissue.^37^

The main challenge of NP decoration via the direct-breath-figure method is the choice of solvent. BFTSA typically employs low-boiling-point solvents that are often difficult to disperse inorganic NPs. To overcome this challenge, surface modifications have been proposed either on NPs^31^ or on the precursor polymeric structure.^38^ Several studies on the generation of amphiphilic or Janus NPs^39−42^ were carried out to enhance the emulsion stability.^30^ This approach results in a proper NP decoration of the edges and the inner bottom areas of the pores.^24,31^ Nevertheless, surface modifications are still solvent-dependent, labor-intensive, and chemistry-specific. To the authors’ knowledge, the literature has yet to offer an alternative, one-stage process that allows selective NP decoration simultaneously during the formation of the pore structures.

In this work, we developed a novel, economical one-stage technique to create a surface porous self-assembled coating, simultaneously guaranteeing NP decoration. The basis of this idea is to implement the conventional BFTSA set up by means of an ultrasonic atomizer, which, by cavitation, can form NP-embedded water droplets. As in the works of Carlomagno *et al.*,^20,21^ a ceramic prosthetic coating was evaluated as a possible application for this technique. A medical-grade UV-cross-linkable alkoxy silicone was used as a PDC precursor to generate the UV-cross-linked BFTSA structure, decorated with synthetized HAp NPs. This organic coating was further transformed into SiON by an organic-to-inorganic pyrolysis process at 900 °C in an NH_3_ environment. Both the NP-decorated polymer structures and the final ceramic structures were physically and chemically characterized.

## 2 Experimental Section

### 2.1 Materials

The silicon preceramic polymer used in this study is Loctite 5248 (AG & Co., Germany, Henkel), a UV-curable alkoxy silicone with thixotropic behavior and a viscosity range of 50–80 cP at 25 °C. The Si/O/C ratio calculated by XPS spectra was 19:27:54 (see Figure S1 in the Supporting Information), while the number-average molecular weight was  = 55 kDa, as measured from static light scattering analysis (SLS, Zetasizer Nano ZEN3600, Malvern Instruments). Loctite 5248, which is a straw-colored one-component liquid solution, was directly dissolved in ethyl acetate (vapor pressure = 73 mmHg at 20 °C; Sigma-Aldrich) to prepare BFTSA precursor solutions at 2 and 5% w/v (mg/mL). The solution was stirred overnight at room temperature and wrapped into an aluminum foil to prevent curing from ambient light. Double-side mechanically polished Ti6Al4V discs of a diameter of 10 mm and a thickness of 2 mm (provided by Eurocoating SpA, Italy) were used as substrates. Calcium nitrate, Ca(NO_3_)_2_ (Sigma-Aldrich, USA); diammonium phosphate, (NH_4_)_2_HPO_4_ (Sigma-Aldrich, USA); gelatin from cold water fish skin (Sigma-Aldrich, USA); deionized (DI) water; and ammonium hydroxide (28%, Millipore, USA) were used to synthesize HAp NPs.

### 2.2 Breath-Figure Setup and Process

The breath-figure setup was adapted from the work of Maniglio *et al.*(43) It consisted of a N_2_ flowmeter (0.4–1.34 L/min), connected to a gas bubbler (water chamber) filled with an NP/DI water (or only DI water) solution. Inside the gas bubbler, an ultrasonic atomizer (a power of 19 W, 1700 ± 50 kHz, and a maximum flow rate of 3000 mm/h) was placed to generate the mist by the cavitation effect. An orbital shaker (KS 125 basic, IKA LABORTECHNIK, Germany) was positioned below the gas blubber to keep the solution properly mixed throughout all the process time. Through a pipe connection, the moisty flow was transported into a 6061 aluminum alloy (MC-Master Carr, USA) reaction chamber, where samples were placed at direct contact with the flow itself. The chamber was sealed with a UV-light transparent PLEXIGLASS Solar, with a hole to allow the casting of the precursor solution by means of a micropipette. Moreover, the setup was equipped with a hygrometer sensor to detect the temperature and relative humidity (RH) inside the reaction chamber. The cross-linking was induced by UV irradiation onto the specimen after a pre-exposure period using a SpotLed365/15 (Photo Electronics Srl, Italy) UV lamp (the emission peak at 365 nm). The ultrasonic atomizer was switched off just before turning on the UV light.

The main process parameters were optimized starting from the works of Carlomagno *et al.*(20,21) and by a trial-and-error approach, supported by optical and electronic microscopy analyses. The process parameters were optimized to obtain a surface porous structure with an equivalent pore diameter ranging between 40 and 250 μm based on a morphological design suitable for cell adhesion^17^ and previous work of Carlomagno *et al.*(21) on biological responses to BFTSA-derived bone prosthetic coatings. Herein, the adopted parameters, either for the direct BFTSA method or for the ultrasonication-assisted BFTSA method, are reported. In the first case, a 5% Loctite 5248–ethyl acetate solution was used with a pre-exposure time (the time after casting the solution and before turning on UV light) of 8 min and a UV exposure time of 5 min. The flow rate and the RH in the chamber were, respectively, set to 0.8 L/min and 99%.

In the second case, a 2% Loctite 5248–ethyl acetate solution and a 5% Loctite 5248–ethyl acetate solution were used. The settings adopted, for both concentrations, were 1 min 30 s of pre-exposure time, 2 min of UV exposure, a flow rate of 1.0 L/min, and a 99% RH. In both cases, 40 μL of Loctite 5248–ethyl acetate solution droplets was used for film casting.

### 2.3 HAp NP Synthesis

HAp synthesis was adapted from the work of Chen *et al.*(44) Aqueous solutions of calcium nitrate (7.84 g in 100 mL of DI water) and diammonium phosphate (3.16 g in 100 mL of DI water) were respectively prepared, and the pH was adjusted to 11 by a dropwise addition of ammonium hydroxide. An aqueous solution of 12 g of gelatin in 200 mL of DI water was prepared and then added to the calcium nitrate solution at 80 °C under vigorous stirring. The diammonium phosphate solution was dropped into the reaction solution at a rate of 2 mL/min using a peristaltic pump (Rainin Dynamax RP-1, Marshall Scientific, USA) under vigorous stirring at 80 °C. Afterward, the solution was left for 2 h at 80 °C before cooling it down to room temperature. It was subsequently centrifuged for 30 min at 4000 rpm (centrifuge, Sigma 2-5, USA) and washed with DI water five times. Finally, the pellet was placed into a 45 °C oven to dry overnight.

### 2.4 Heat Treatment

The heat treatment was performed on the polymeric BFTSA specimens (Hap-decorated and nondecorated samples) to induce the organic-to-inorganic conversion. As described in Section 3.2, also, HAp NPs alone had been heat-treated with the same procedure to evaluate if any changes could occur to the NPs. The heat treatment was performed in a silica tubular furnace (Thermo Scientific Heraeus) at 900 °C in an NH_3_ atmosphere. A purging time of 1 h at room temperature was promoted before starting the heating cycle. The cycle adopted consisted of a heating ramp until 120 °C at 5 °C/min, after which it was held for 2 h at 120 °C. Subsequently, a further heating ramp, still at 5 °C/min, was promoted to reach 900 °C. After holding for 1 h at 900 °C, a cooling ramp at 5 °C/min was set to go back to room temperature. The NH_3_ flow was set at 0.4 L/min (LPM).

### 2.5 Characterization

HAp NPs were characterized by scanning electron microscopy (SEM), FESEM, (Zeiss supra 40, Germany), and transmission electron microscopy (TEM; Talos F200 S G2, Thermo Fisher Scientific, USA) to evaluate their size and morphological features. X-ray diffraction (XRD) analysis with an IPD 3000 (scan from 5° to 120°, Co kα = 1.7902 Å radiation, scan step = 0.03°, and an applied voltage and a current of 40 V and 30 mA, respectively) was performed to determine the HAp structure before and after the pyrolysis stage in an ammonia environment. XRD raw data were then elaborated by MAUD, XRD software based on Rietveld refinement. The patterns of the reference phases were supplied by the crystallography open database (COD). The surface and morphology of the coating, either in the polymeric stage or in the ceramic stage, were inspected by FESEM and by energy-dispersive X-ray spectrometry (EDXS) microanalysis, with a Jeol IT300 equipped with a Bruker EDXS. The latter had also been performed to obtain a semiquantitative analysis of the chemical composition of the coating and to detect the NP presence by mapping the calcium and phosphorus elements. All the specimens were previously coated with a platinum/palladium (Pt/Pd, 80:20) conductive thin film (Q150T ES, Quorum Technologies, UK) before the FESEM and EDXS analyses. For XPS, an Axis DLD Ultra (Kratos—UK) analyzer equipped with a monochromatized Al kα X-ray source of 1486.6 eV was used to evaluate the chemical composition and the bonding state of the ceramic coatings. Spectra were analyzed with homemade software based on the R platform.^45^ Sessile contact angle (CA) measurements were carried out to evaluate the surface physical properties of the BFTSA specimens after ceramic conversion. CA analyses were performed with a homemade equipment and a 3 μL DI water droplet.

### 2.6 Statistical Analysis

Statistical analyses were conducted to study the pore size and its distribution as well as the effect of the Pickering emulsion. The pore analyses were carried out using ImageJ by thresholding techniques on the SEM images. Pore area values were obtained from different images of the same specimen and then analyzed with Origin Pro Lab. Histograms, showing the pore area distribution, were generated with a bin size ranging from 600 to 800 μm^2^. The distributions of the pore size were analyzed using a log–normal function for the more regular areas and an exponential function for regions where the coalescence dominated. For the Pickering emulsion evaluation, a perfect circularity was assumed to express the equivalent diameter values, which have a more direct understanding of the pore dimensions. Concerning the pore size statistical analyses, the median, quartiles (Q1 and Q3), and interquartile range (IQR) were reported. Circularity tests were performed using the above-mentioned software and a box chart diagram has been reported, showing the trend as function of an increase of the HAp content in the specimen.

## 3 Results and Discussion

### 3.1 Breath-Figure Structures

**Figure 1:**
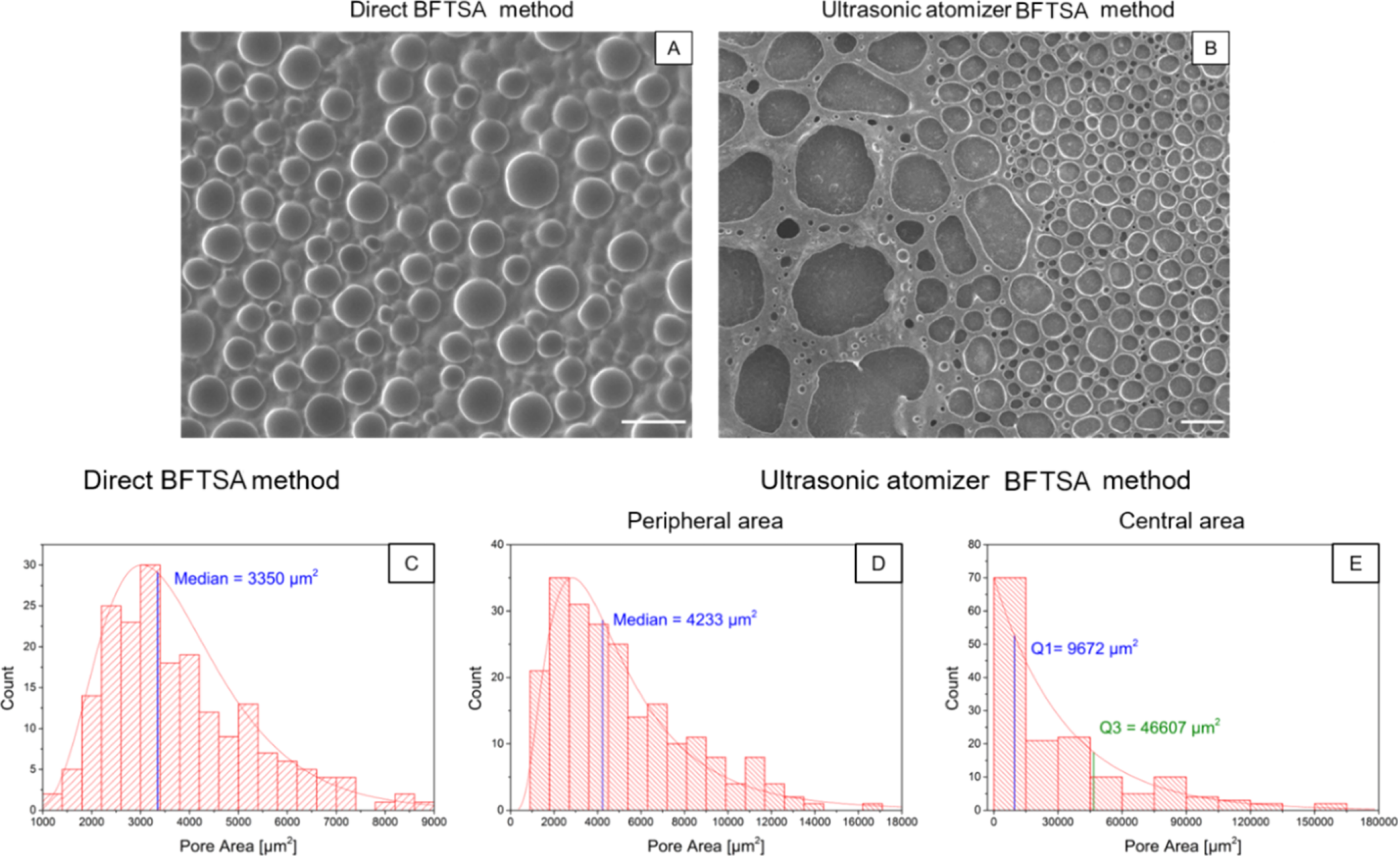
Comparison between the direct BFTSA and ultrasonic atomizer BFTSA methods. (A,B) SEM images of samples obtained from the direct BFTSA method and from the ultrasonic atomizer BFTSA method (scale bars: 100 μm). (C–E) Histograms and pore area distribution of structures as labeled.

**Figure 2:**
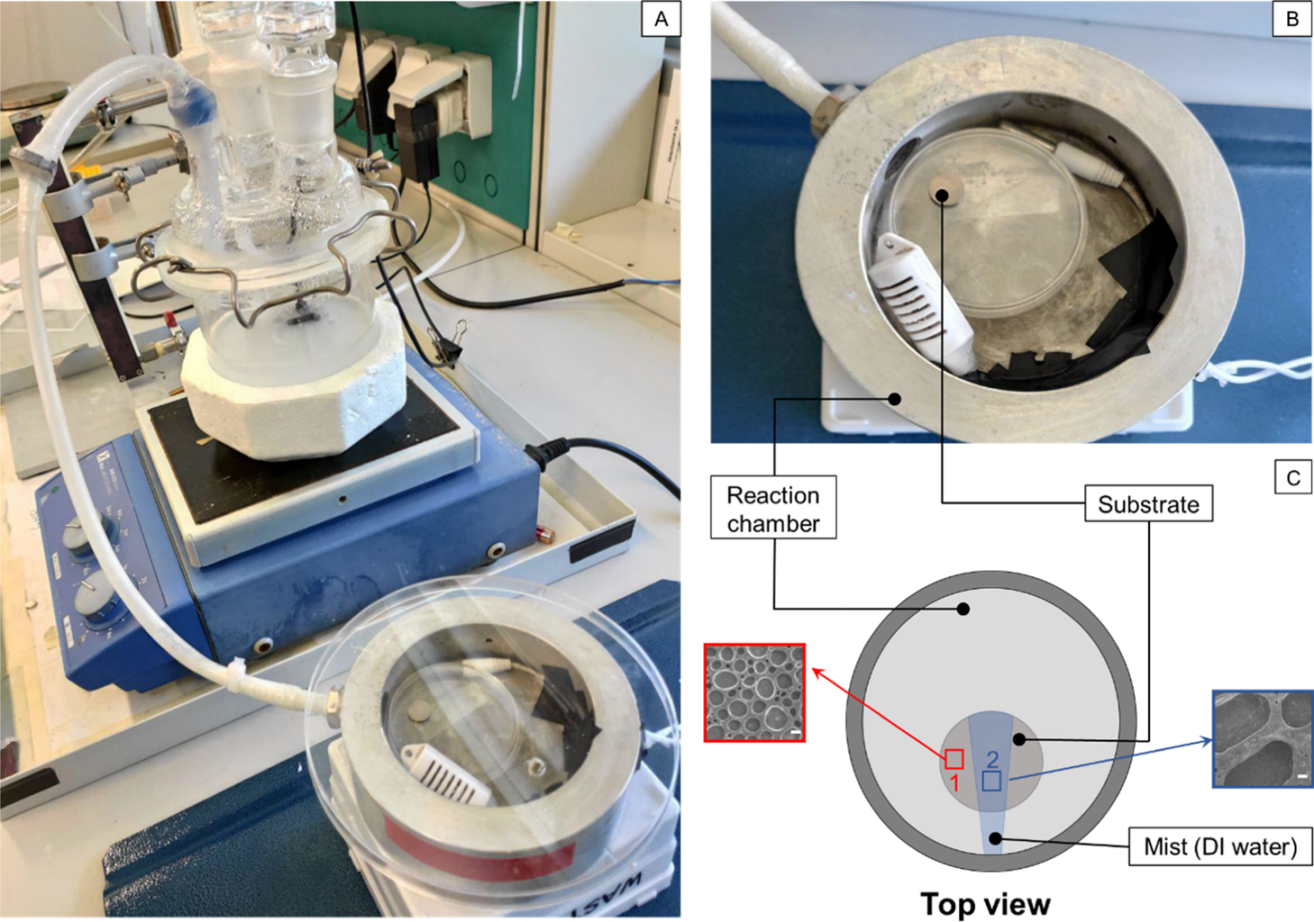
3D (A) and top-view image (B) of the chamber setup with the sample placed inside. In (C), the top-view sketch of the chamber is reported, highlighting the two generated BFTSA regions with different morphological structures. The SEM image in the blue square is referred to the area directly hit by the mist flow, and the one in the red square is instead the peripheral area of the substrate. Scale bar: 100 μm.

Breath-figure structures, obtained with the method reported in Section 2.2 using a solution of 5% (w/v) Loctite 5248–ethyl acetate, have been investigated and compared to specimens obtained via the direct-breath-figure method. The latter is based on the experiments described in the work of Carlomagno *et al.*(20) As reported in Figure 1A,B, a morphological comparison of the obtained patterns was carried out by a statistical analysis of the SEM images (Figure 1C–E) to determine the pore areas and their distribution. The main difference between the two adopted BFTSA methods lies in the central areas of the specimens where the mist flow generated by the ultrasonic atomizer directly impacts the casted Loctite 5248–ethyl acetate solution. As illustrated in Figure 2, two different regions of pore structures were observed: a peripheral region (red box, number 1) with smaller and more regular pore sizes and a central region (blue box, number 2) with bigger and less spherical pores. The differences in pore morphology and dimension are related to two distinct physical mechanisms that occur on the specimen. In the central region, water droplet condensation is also assisted by a water droplet deposition onto the polymeric solution because of the mist flow produced by the ultrasonic atomizer. Collectively, the two phenomena lead to a more pronounced coalescence, which promotes the formation of larger and less regular pores.^2,4^ On the other hand, in the peripheral region, the droplet formation mechanism is dominant by condensation of water droplets, induced by the humid environment in the reaction chamber. This hypothesis is supported by the histograms reported in Figure 1C–E. The peripheral region shows a log-normal distribution (see Figure 1D), similar to that obtained by the direct BFTSA method (Figure 1C), while the central region shows an exponential distribution (Figure 1D).

Concerning the physical mechanisms that lead to the BFTSA structures, it is also necessary to take into consideration the wetting/dewetting phenomena. Dewetting can occur at a solid–liquid interface (i.e., titanium–Loctite 5248–ethyl acetate solution), when spontaneous spreading of the liquid is not developed and liquid withdrawal and retraction are observed on the surface.^46^ In this context, it is worth noting that dewetting of the Loctite 5248–ethyl acetate solution during the pore-forming process was not observed as both the solute and solvent are of low surface tension. As a matter of fact, complete spreading of the solution on the high-energy substrate occurs during all experiments. Furthermore, local dewetting within the large coalesced pores was also not relevant as XPS measurements (see Section 3.4) of the converted inorganic coating only show ∼1% Ti. Therefore, regardless of the size and shape of the pores formed, the coating layer covers the entire substrate instead of forming a gridlike network structure due to local dewetting. Furthermore, photo cross-linking during the BFTSA process hinders the mobility of growing polymer chains and generates elastic forces that suppress the dewetting tendency.^47^ Combining the abovementioned factors, it is unlikely that dewetting plays any role in the pore structure formation in the BFTSA process.

As given by the histogram in Figure 1D, the median value of the fitted curve is of 4233 μm^2^, while the quartiles (Q1 and Q3) and the IQR are, respectively, 2768, 6474, and 3706 μm^2^. The histogram in Figure 1E instead reports a larger population in a small range of values, as deducible from Q1 (9672 μm^2^) and Q3 (46,607 μm^2^), showing 25% (blue area) and 75% (blue + green area) of the measurements. Considering the statistical analyses performed on the entire specimen, the exponential decay is the prevailing distribution with a Q1 and a Q3 of 4665 and 22,480 μm^2^, respectively. These structures were produced with the parameters reported in Section 2.2, accurately chosen to reach a pore dimension suitable for a prosthetic coating application, as reported in ref (17). The following discussion regards the analyses of the more regular regions because these are the prevalent areas of the samples. Furthermore, to increase regularity, specimens were placed off the center of the reaction chamber because in the center of the reaction chamber, the direct droplet deposition and the corresponding coalescence are more pronounced.

### 3.2 HAp NPs

**Figure 3:**
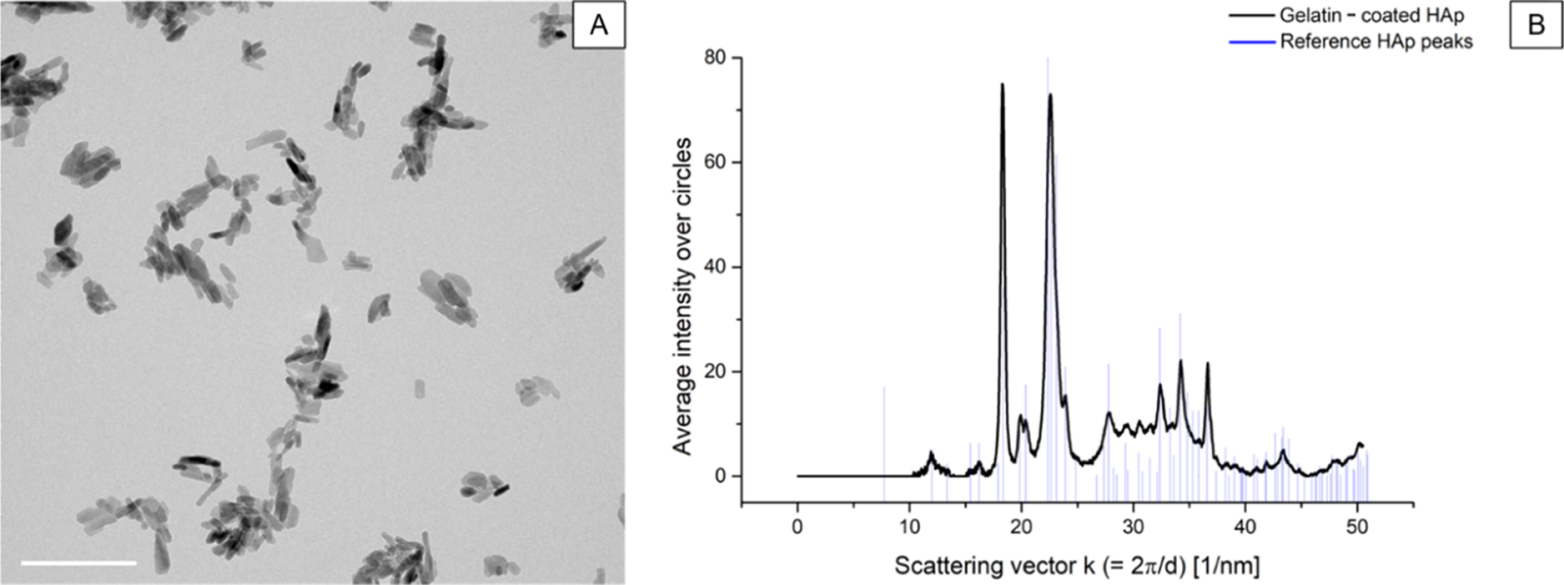
(A) TEM image of the rodlike shape of the HAp NPs. The scale bar is 200 nm. (B) X-ray diffractogram obtained by SAED analysis in comparison with the reference HAp peaks.

TEM analysis reveals that HAp NPs have a rodlike morphology with dimensions of 52 ± 22 and 16 ± 6 nm (Figure 3A). As given in the diffractogram of Figure 3B, obtained from the selected area electron diffraction (SAED) analysis, the peak matching between the NP structure and a reference HAp spectrum reported in the literature was confirmed.^48^ The synthesis route also involved a gelatin coating in order to increase the dispersibility and stability of HAp in DI water, which nevertheless did not affect the crystalline structure of the HAp NPs. For a more detailed particle characterization, FT-IR, TGA, and DTA are provided in the Supporting Information (Figure S2).

**Figure 4:**
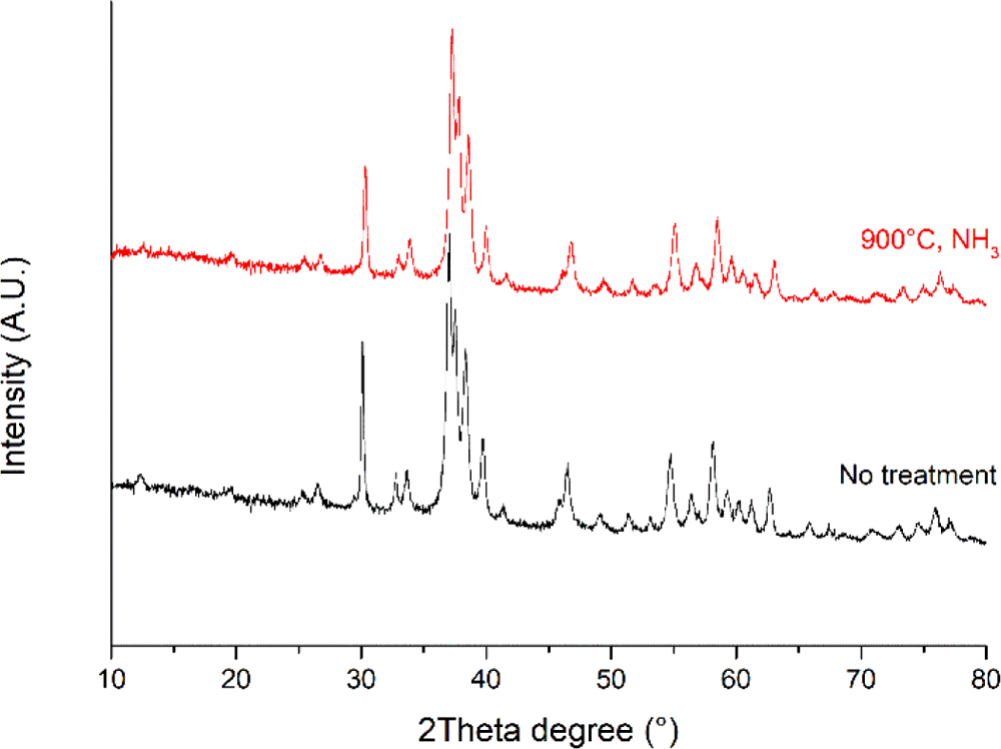
XRD spectra of the as-synthesized HAp nanopowder (black spectrum) and the NH_3_ heat-treated HAp nanopowder (red spectrum).

HAp NPs were also pyrolyzed at 900 °C in an NH_3_ environment for 1 h to evaluate if any reaction could have occurred once implemented in the BFTSA structures. As a matter of fact, NH_3_ heat treatment of HAp NPs was performed to provide the environment for organic-to-inorganic transformation as the BFTSA samples. Accordingly, XRD analysis was performed on the HAp nanopowder before and after the heat treatment. In Figure 4, the comparison between the two XRD spectra is reported, showing no changes in the HAp structure, neither due to the NH_3_ environment nor due to the process temperatures, and both spectra match data reported in COD-ID: 4317043 (HAp).

A HAp NPs–DI water suspension at 20% (w/v) was then produced and introduced into the water chamber via the atomizer in order to evaluate the NP transport into the reaction chamber.

**Figure 5:**
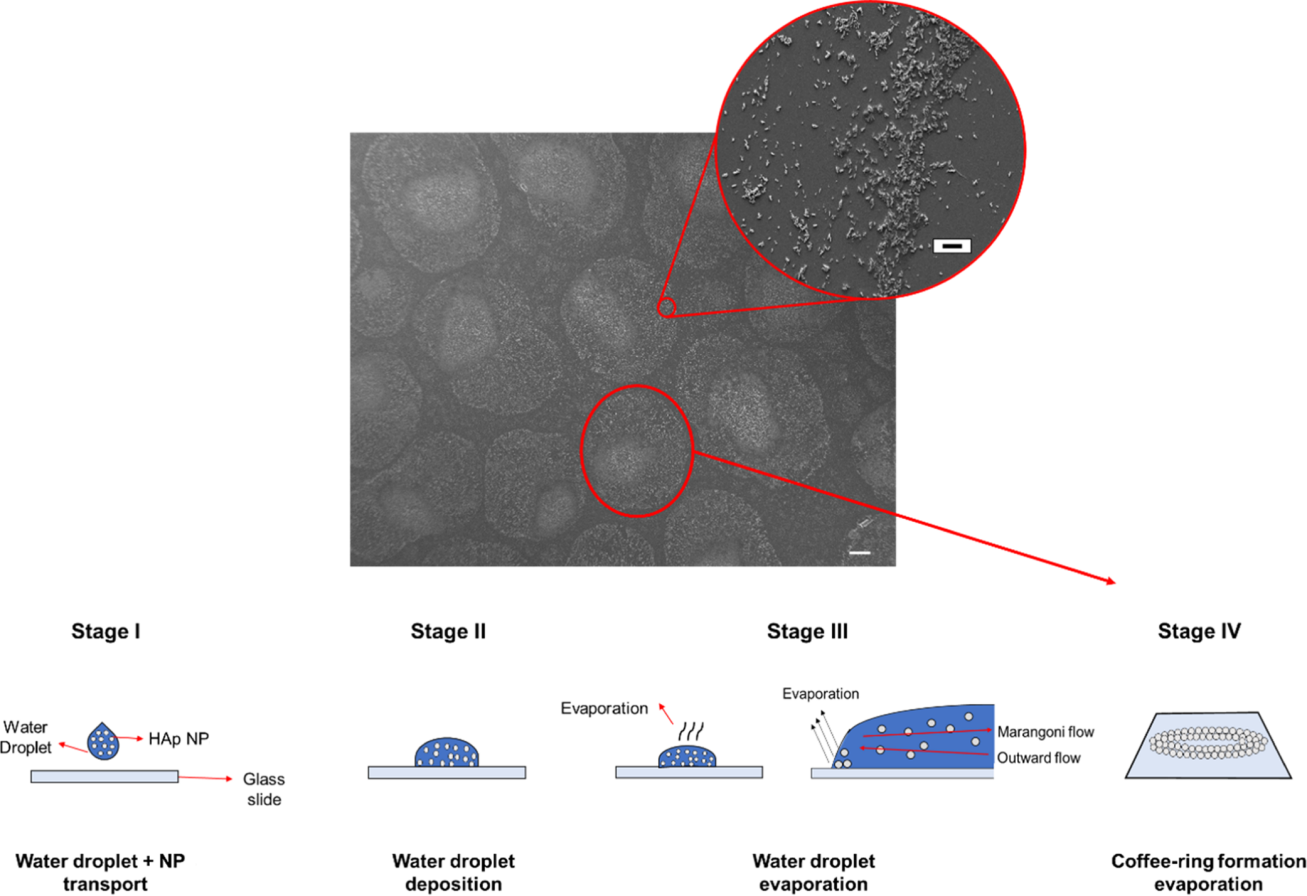
FESEM images of the HAp NP deposition onto a glass substrate in a coffee-ring-like shape. The scale bar is set to 100 μm for the main SEM image and 4 μm for the magnified view. The coffee-ring-like mechanism can be divided into four different stages, namely, droplet transport, droplet deposition, water evaporation, and coffee-ring formation, as depicted in the sketches.

The tests revealed that NPs were successfully deposited, without affecting the deposition time of the process. As a matter of fact, varying the deposition time, coffee-ring-like particle deposition was always observed. Each ring represents the trace of a coalesced droplet prior to the drying, as shown in Figure 5.

These coffee-ring structures show an assembly of HAp NPs into multiple layers at the edges of the evaporated water droplets. In addition, NP depositions at the inner part of the droplet are observed. The overall NP distribution within each isolated region is inhomogeneous, which is attributed to both the coalescence of droplets during droplet formation and the subsequent water evaporation. First, significant droplet coalescence is expected from both direct droplet deposition from the mist flow and condensation, which results in the overall droplet shape and corresponds to the overall trace of the NP deposition. Second, within each droplet trace, the distribution of NPs is dictated by the energy minimization mechanism and transport behavior during water evaporation. Particularly, the enhanced deposition at the droplet edge is by a combination of the transport of NPs to, and stabilization of NPs at the three-phase contact line where the water evaporation rate is the highest. Similar to a typical BFTSA process, the Marangoni flow associated with the temperature-induced surface tension gradients will carry and deposit some NPs toward the droplet center.^49^ Such a nonuniform deposition pattern, often considered a limitation for coffee-ring applications,^49^ could be adopted to selectively decorate edges and the inner part of pores. Nevertheless, such initial analysis confirms that the home-built setup is suitable for the proposed ultrasonication-assisted BFTSA method.

### 3.3 Decorated BFTSA Structures and the Pickering Emulsion Effect

**Figure 6:**
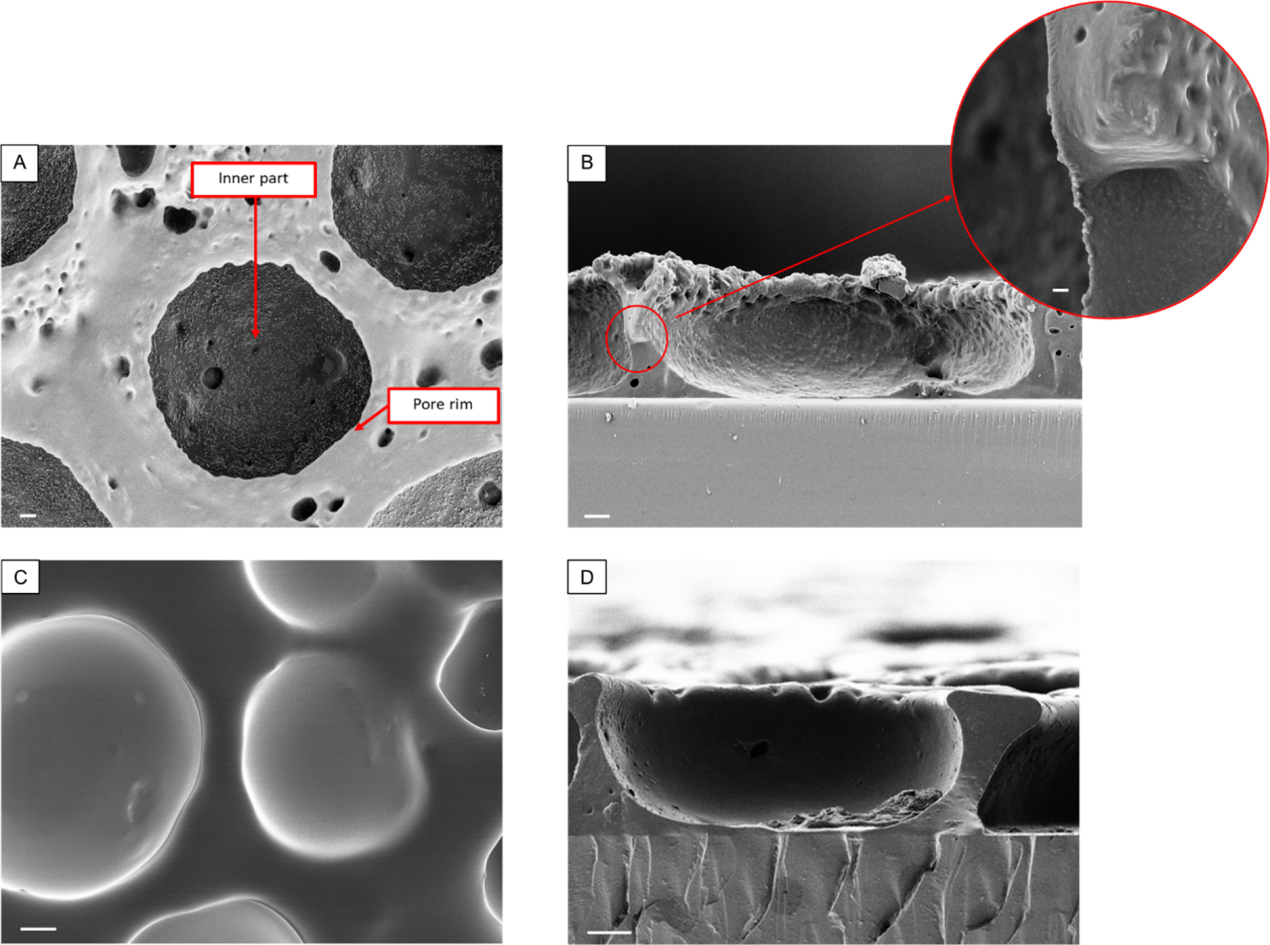
FESEM images of the NP-decorated BFTSA structures at the 5% Loctite 5248–ethyl acetate solution and 20 mg/mL HAp/DI water. (A) Top-down view on the Ti6Al4V disc substrate. (B) Cross-sectional view on a glass substrate. (C,D) Top-down and the cross-sectional views of the BFTSA structure in the absence of NP decoration. The scale bars are set to 10 μm for (A–D) and to 1 μm for the magnified view in (B).

The process variables investigated to optimize the NP decoration of the BFTSA structures are reported in Table 1. Two parameters have been evaluated as principal factors affecting the process, namely, Loctite 5248 concentration and HAp amount in the DI water solution. Having a sufficiently high precursor concentration is important to avoid the gravity-driven sedimentation of the particles. Increasing concentration from 2 to 5% leads to an overall increase of the precursor solution viscosity, thus reducing NP mobility prior to the cross-linking stage and therefore keeping the particles on the surface and limiting the embedment into the matrix. In addition, the 5% solution is expected to reach gelation during the evaporation/cross-linking quicker than the 2% solution, which results in a reduced time for NP sedimentation and often formation of a denser skin layer.^50^ On the other hand, the increment of concentration of HAp NPs in the gas bubbler results in NP-enriched droplets, which will likely increase the level of pore decoration. Different HAp concentrations were therefore inspected, varying from an initial 1.5 mg/mL to 5 mg/mL and eventually to 20 mg/mL. The optimum BFTSA structure decoration was obtained with a 5% (w/v) Loctite 5248–ethyl acetate solution and a 20% (w/v) HAp NPs–DI water solution. Accordingly, FESEM images of the obtained structures are shown in Figure 6A,B.

**Table 1:** Process Parameters Inspected to Optimize the NP Embedment into the BFTSA Structures

parameters	conditions
Loctite 5248	2%/5% (w/v)
concentration of HAp in DI water	0/1.5/5/20 mg/mL

As expected, similar to Figure 5, the decoration mainly occurred in the inner part of the pores (Figure 6A) and at the three-phase (air–water–polymer) contact (cross-sectional view in Figure 6B); these results are also in accordance with the work of Sun *et al.*(24) and Yang *et al.*(31)

The effect of the increasing amount of HAp NPs on the pore morphology, regularity, and circularity was also investigated by comparing 5% Loctite 5248 samples with no HAp NPs and 1.5, 5, and 20 mg/mL NP concentrations. In Table S1 (see the Supporting Information), the statistical data of the pore areas and the circularity analysis are reported for the four samples. Furthermore, the equivalent diameter (ϕ_eq_) is reported under the hypothesis of perfect circularity of the pores.

**Figure 7:**
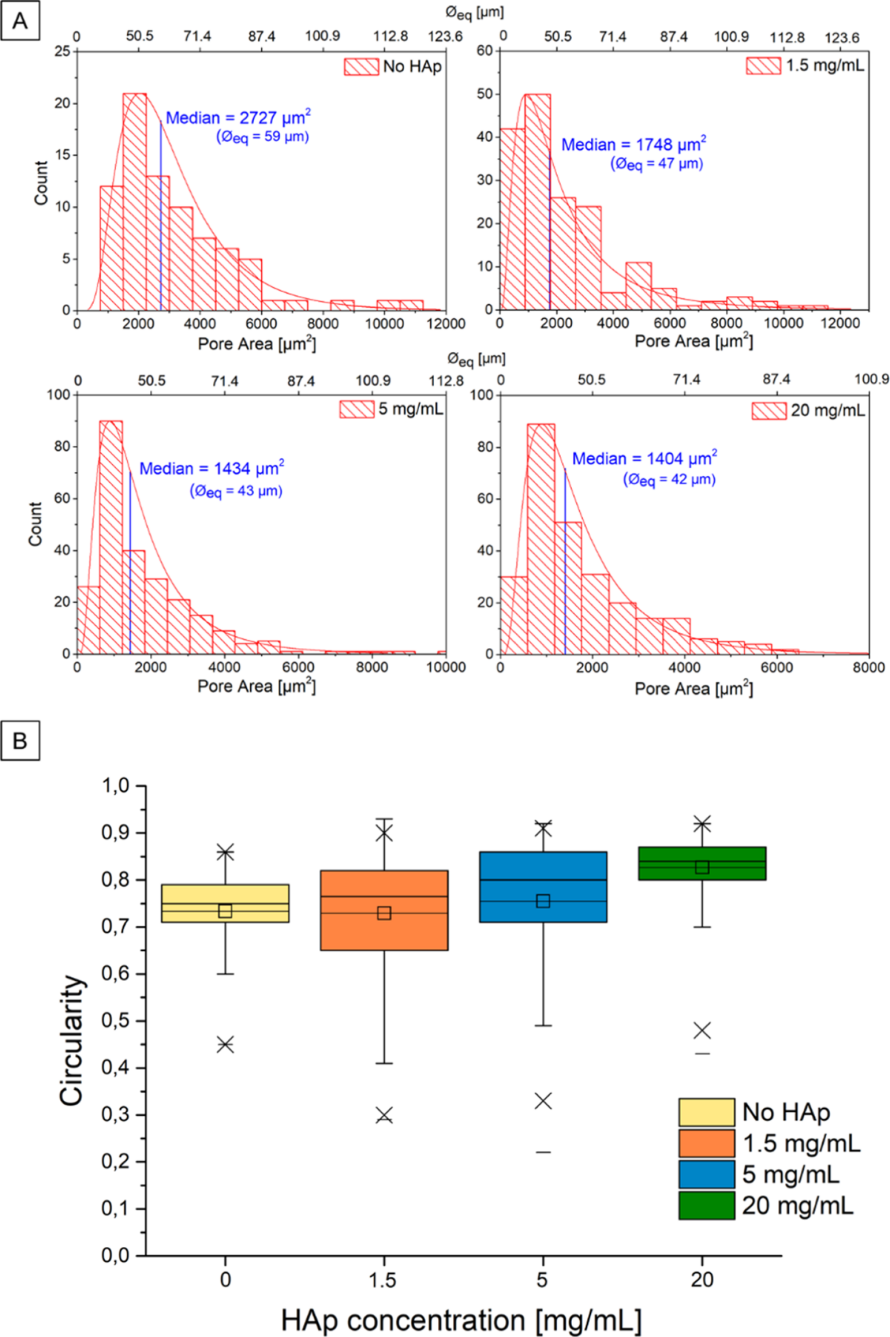
(A) Pore area distribution histograms with log–normal fitting and the median value for the four tests at different HAp concentrations. (B) Box chart of the circularity statistical values related to the HAp concentration (mg/mL) for the same four tests reported in Table S1.

As given in Table S2 and Figure 7A, a decrease of the median value is noticeable from the sample with no HAp (2727 μm^2^ or ϕ_eq_ = 59 μm) to the one with 20 mg/mL HAp (1404 μm^2^ or ϕ_eq_ = 42 μm). The most relevant decrease is between the first two samples (no HAp vs 1.5 mg/mL), moving from 2727 (ϕ_eq_ = 59 μm) to 1748 μm^2^ (ϕ_eq_ = 47 μm), while it slows down with a further NP increase. On the contrary, for the circularity analysis, an opposite trend is observed: the higher the NP concentration, the higher the pore circularity, toward the asymptotic value of 1 (a perfect circle). A similar trend is observed for Q1, median, and Q3 values, which increase, respectively, from 0.71, 0.75, and 0.79 for the sample with no HAp to 0.80, 0.84, and 0.87 for the sample with 20 mg/mL HAp. Furthermore, a noticeable decrease of IQR with an increase of NP concentration is observed, which indicates that the distribution becomes narrower (Table S2 and Figure 7B). This is more evident from 5 mg/mL (IQR = 0.17) to 20 mg/mL (IQR = 0.07) because the IQR is almost half of the previous one. The circularity increase is however limited, and it is more pronounced for concentrations from 1.5 to 5 mg/mL, where the median values increase from 0.76 up to 0.80, than for concentrations from 5 to 20 mg/mL, where the median increases from 0.80 to 0.84. From these data, particle decoration for low concentrations is more effective in limiting the pore dimension, while for higher concentrations, NPs affect the pore circularity more.

### 3.4 Ceramic Conversion

**Figure 8:**
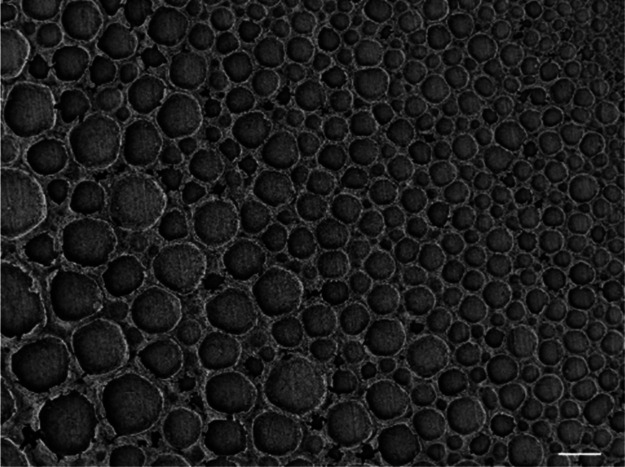
SEM image of a SiON BFTSA structure at 5% Loctite 5248–ethyl acetate. Few coating cracks visibly connect the pore rims. The scale bar is set to 100 μm.

The NH_3_ pyrolytic transformation of the decorated BFTSA preceramic structures was performed according to the procedures described by Carlomagno *et al.*,^20^ which transforms a cross-linked Si-based polymer precursor into a SiON structure. The NH_3_ environment had been chosen in line with a further work of Carlomagno *et al.*,^21^ where these structures, compared to SiO_2_ and SiOC coatings, are more suitable to promote cell adhesion, proliferation, and activity. The ceramic conversion has been evaluated by SEM, EDXS, and XPS analyses. SEM images of the SiON samples had been collected to evaluate the overall macrostructure. As shown in Figure 8, the surface porous structure has been preserved without any evident morphological changes as well as signs of significant crack propagation or delamination. A predictable shrinkage occurred because of the pyrolysis, as similarly reported in ref (20), which nevertheless does not significantly alter the morphological features of the parent polymeric BFTSA structures.

**Figure 9:**
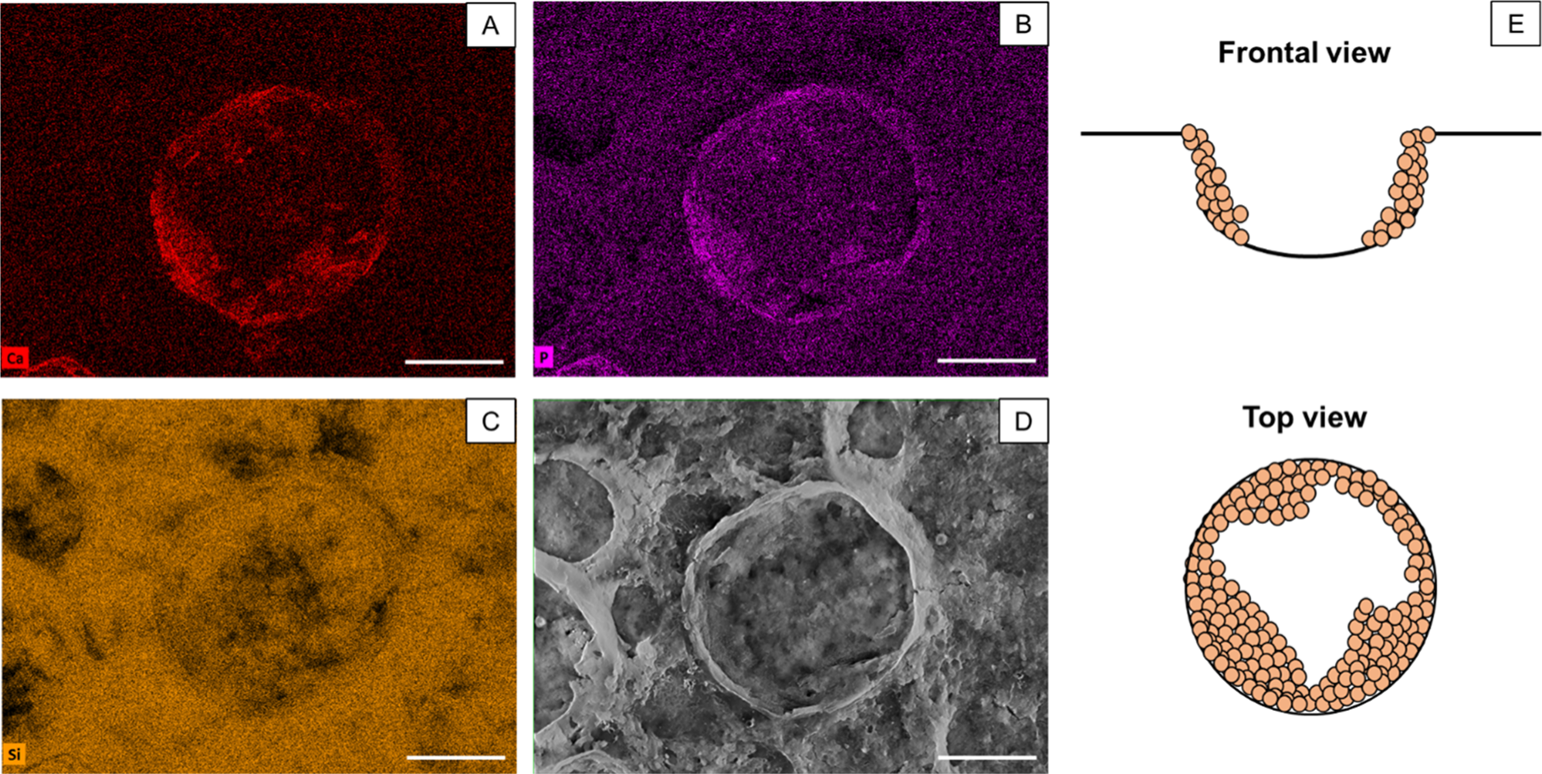
EDXS elemental maps for calcium (A), phosphorous (B), and silicon (C). The SEM raw image of the mapped pore (D). The scale bars are 100 μm. (E) shows schematics of the pore decoration from a frontal view and a top view.

EDXS elemental maps were acquired to semiquantitatively determine the spatial distribution of Si, Ca, and P in the inorganic coatings and to detect the incorporation of NPs into the structure. From Figure 9, where the Ca (Figure 9A), P (Figure 9B), and Si (Figure 9C) maps are reported, it is clear that the HAp NPs were decorated along the interface and the inner part of the pores, given by a higher intensity of both the Ca and P signals, while the Si signal is more homogeneous across the entire image, indicating that the matrix is mainly characterized by Si, as expected from the conversion into SiON.

Nevertheless, because of the limitations and the reliability of the EDXS technique to detect light elements such as oxygen and nitrogen, XPS was performed to quantitatively determine the chemical composition and the chemical bonds generated during the organic-to-inorganic transformation. Two different specimens were analyzed: 5% Loctite 5248 with HAp NPs and 5% Loctite 5248 without HAp NPs.

**Figure 10:**
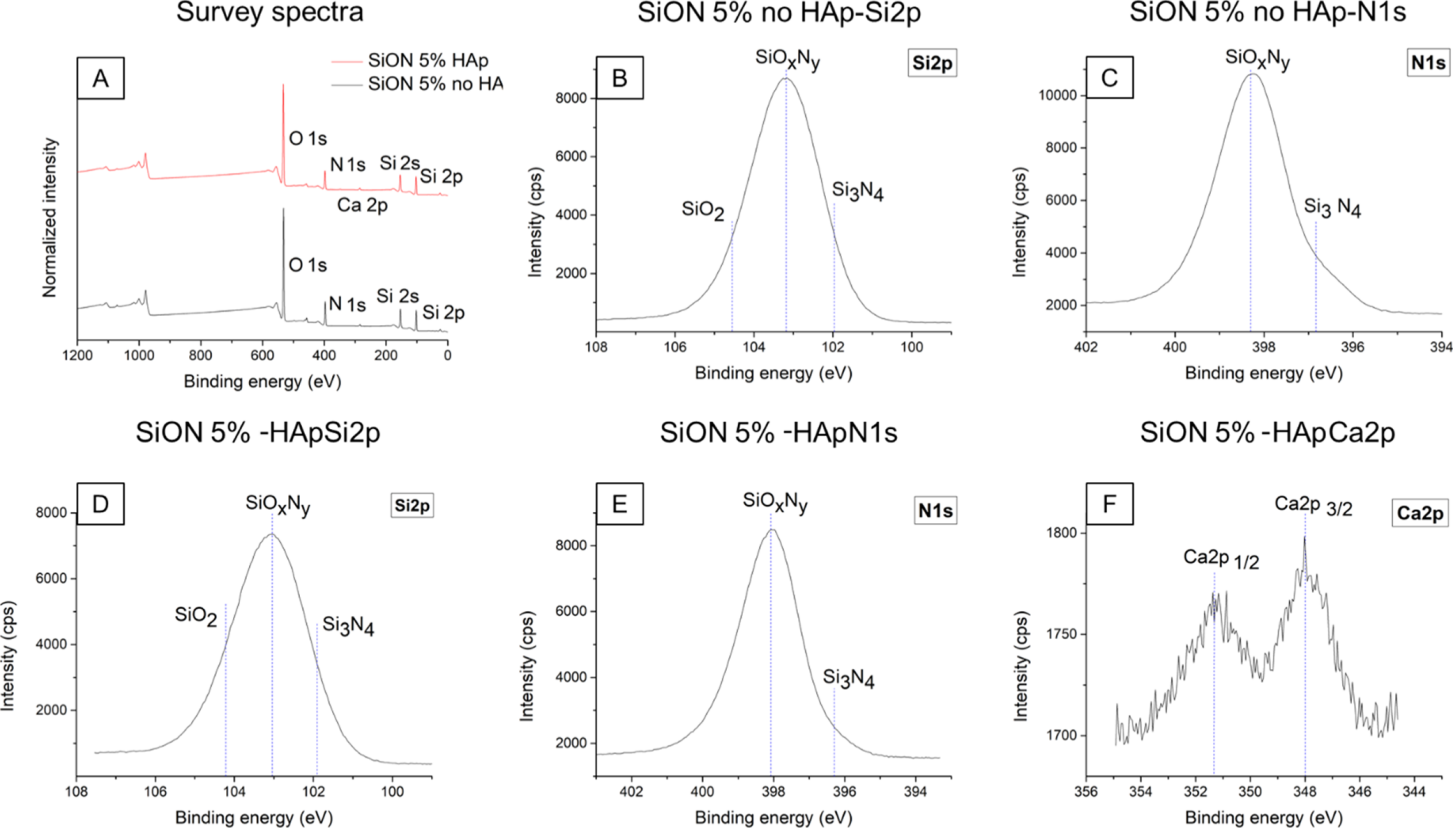
(A) XPS survey spectra for NH_3_-pyrolyzed BFTSA samples of a 5% Loctite 5248–ethyl acetate solution with (black spectrum) and without HAp NPs (red spectrum). (B) Si 2p and (C) N 1s spectra for the SiON 5%–no HAp sample; (D–F) Spectra of Si 2p, N 1s, and Ca 2p of the SiON 5%–HAp sample.

The survey spectra of the two tested samples are shown in Figure 10A. As expected, in both spectra, Si 2p, O 1s, and N 1s peaks display the highest intensity, confirming the successful SiON conversion. The calculated atomic percentages for Si 2p, O 1s, and N 1s are, respectively, 27, 50, and 18% for the sample without HAp and 24, 54, and 16% for the heat-treated sample with HAp NPs. In both spectra, the C 1s peak is also visible at a binding energy of 285.10 eV and it has been attributed to an adventitious hydrocarbon contamination of the samples. The main difference between the two spectra is given by the Ca 2p peak revealed at 348.02 eV for the SiON 5%–HAp specimen, suggesting the HAp NP presence into the BFTSA structure. However, the low intensity of the Ca 2p spectrum observed can be attributed to a masking effect caused by the presence of particles of small dimensions onto the inner walls of the pores. Nevertheless, as shown in Figure 10F, the typical Ca 2p doublet is reported, with the two spin–orbit splitting component peaks at 351.34 eV (Ca 2p_1/2_) and at 348.00 eV (Ca 2p_3/2_). These binding energy values are compatible with Ca in HAp.^51−53^ These values are also in agreement with other HAp XPS analysis results reported in the literature.^54−56^ From the Si 2p deconvoluted spectra for both specimens, reported in Figure 10B,D, three components are present, SiO_2_, Si_3_N_4_, and SiO_*x*_N_*y*_, of which the last is the prevailing component with a peak at 103.18 eV (Figure 10B) and 103.04 eV (Figure 10D). This trend is also confirmed by the deconvoluted spectra of N 1s in Figure 10C,E, where the main contribution is still given by SiO_*x*_N_*y*_, reported at 398.30 and at 398.09 eV, respectively, while the second peak is related to Si_3_N_4_ (i.e., at 396.84 and 396.30 eV). The obtained values are in good agreement with the reported literature^57−60^ and with the previous work of Carlomagno *et al.*,^20^ conducted on the same polymeric precursor under identical pyrolysis conditions. Similarly, the wettability characteristics of flat and surface porous BFTSA structures were studied by means of static CA analysis. Nonpatterned surfaces evidenced a marked hydrophilic behavior (CA = 24 ± 2°), while surface porous BFTSA evidenced complete spreading of the water droplets. This behavior can be attributed to capillary phenomena deriving from small coating cracks (also visible in the SEM image, Figure 8) produced by the pyrolysis conversion of the BFTSA specimens.

SEM, EDXS, and XPS analyses confirmed the ceramic conversion of the polymeric material as well as the preservation of the surface porous structure at a macroscale and a microscale. Furthermore, HAp NPs were detected and also characterized on the ceramic samples, stating, as expected, that the heat treatment did not provide any change in the structure characteristics.

## 4 Conclusions

Breath-figure surface porous structures are suitable for a wide range of applications. Particularly, it has been demonstrated that using a Si-based preceramic polymer as a precursor, it is possible to obtain inorganic coatings suitable for prosthetic coating applications.^21^ Furthermore, by selectively decorating these pores with NPs, a further enhancement of osteointegration can be achieved. Nevertheless, the NP embedment into the BFTSA structures might require the need to surface-modify particles or the precursor by highly demanding, time-consuming, and expensive processes.^24,25,31^

In the present study, a novel, economic one-stage technique to combine the BF formation together with the NP pore decoration is proposed. This is achieved by introducing an ultrasonic atomizer into the gas bubbler in order to directly deposit particles on the casted polymeric solution by mist transport. Adopting this new method, this study demonstrates the successful formation of coatings for prosthetic applications using Loctite 5248, a UV-cross-linkable PDC alkoxy silicone, and synthesized HAp NPs. NP decoration occurred at the three-phase interface and into the inner bottom area of the BFTSA pores. It has also been noticed that by increasing the HAp decoration, beneficial effects regarding the circularity of the patterned pores were achieved, in accordance with the combination of Pickering emulsion and BF mechanisms. Once the organic structures were characterized, a pyrolytic process, in an NH_3_ atmosphere, at 900 °C for 1 h was promoted. The inorganic transformation into SiON successfully occurred, together with successful decoration of HAp NPs, as confirmed by the XPS and EDXS measurements. As confirmed by SEM analysis, the overall morphological features of the coatings have not changed after the heat treatment, preserving the typical BFTSA structure.

Future developments, currently under investigation, can be related to embed other types of functional NPs, for example, drug-loaded mesoporous NPs, to further boost the osseointegration or to use the coating as a drug delivery scaffold. Further investigations concerning the biological response of these coatings are also underway.

## Supporting Information Available

Loctite chemistry and XPS analysis spectra; HAp NP further characterization (FT-IR, TGA, and DTA); SEM images of HAp-decorated BFTSA structures; statistical data related to Pickering evaluation (PDF)

## Abbreviations

BF: breath figure
BFTSA: breath-figure-templated self-assembly
CA: contact angle
EDXS: energy-dispersive X-ray spectrometry
FESEM: field emission scanning electron microscopy
HAp: hydroxyapatite
LPM: liter per minute
NP: nanoparticle
PDC: polymer-derived ceramic
SAED: selected area electron diffraction
SLS: static laser scattering
TEM: transmission electron microscopy
XPS: X-ray photoelectron spectroscopy
XRD: X-ray diffraction

## References

[ref1] Huie J. C. Guided Molecular Self-Assembly: A Review of Recent Efforts. Smart Mater. Struct. 2003, 12, 264–271. 10.1088/0964-1726/12/2/315.

[ref2] Zhang A.; Bai H.; Li L. Breath Figure: A Nature-Inspired Preparation Method for Ordered Porous Films. Chem. Rev. 2015, 115, 9801–9868. 10.1021/acs.chemrev.5b00069. 26284609

[ref3] Rayleigh L. Breath Figures. Nature 1911, 86, 416–417. 10.1038/086416d0.

[ref4] Dou Y.; Jin M.; Zhou G.; Shui L. Breath Figure Method for Construction of Honeycomb Films. Membranes 2015, 5, 399–424. 10.3390/membranes5030399. 26343734 PMC4584288

[ref5] Bormashenko E.; Musin A.; Bormashenko Y.; Whyman G.; Pogreb R.; Gendelman O. Formation of Films on Water Droplets Floating on a Polymer Solution Surface. Macromol. Chem. Phys. 2007, 208, 702–709. 10.1002/macp.200600485.

[ref6] Nepomnyashchy A. A.; Golovin A. A.; Tikhomirova A. E.; Volpert V. A. Nucleation and Growth of Droplets at a Liquid-Gas Interface. Phys. Rev. E: Stat., Nonlinear, Soft Matter Phys. 2006, 74, 021605 10.1103/PhysRevE.74.021605. 17025444

[ref7] Zhang J.; Sun B.; Huang X.; Chen S.; Wang G. Honeycomb-like porous gel polymer electrolyte membrane for lithium ion batteries with enhanced safety. Sci. Rep 2014, 4, 6007 10.1038/srep06007. 25168687 PMC4148667

[ref8] Bormashenko E.; Pogreb R.; Stanevsky O.; Bormashenko Y.; Socol Y.; Gendelman O. Self-Assembled Honeycomb Polycarbonate Films Deposited on Polymer Piezoelectric Substrates and Their Applications. Polym. Adv. Technol. 2005, 16, 299–304. 10.1002/pat.585.

[ref9] Kurono N.; Shimada R.; Ishihara T.; Shimomura M. Fabrication and Optical Property of Self-Organized Honeycomb-Patterned Films. Mol. Cryst. Liq. Cryst. 2002, 377, 285–288. 10.1080/713738506.

[ref10] Zhu Y.; Sheng R.; Luo T.; Li H.; Sun J.; Chen S.; Sun W.; Cao A. Honeycomb-Structured Films by Multifunctional Amphiphilic Biodegradable Copolymers: Surface Morphology Control and Biomedical Application as Scaffolds for Cell Growth. ACS Appl. Mater. Interfaces 2011, 3, 2487–2495. 10.1021/am200371c. 21699231

[ref11] Rungsiyakull C.; Li Q.; Sun G.; Li W.; Swain M. V. Surface Morphology Optimization for Osseointegration of Coated Implants. Biomaterials 2010, 31, 7196–7204. 10.1016/j.biomaterials.2010.05.077. 20573394

[ref12] Gittens R. A.; McLachlan T.; Olivares-Navarrete R.; Cai Y.; Berner S.; Tannenbaum R.; Schwartz Z.; Sandhage K. H.; Boyan B. D. The Effects of Combined Micron-/Submicron-Scale Surface Roughness and Nanoscale Features on Cell Proliferation and Differentiation. Biomaterials 2011, 32, 3395–3403. 10.1016/j.biomaterials.2011.01.029. 21310480 PMC3350795

[ref13] Mavrogenis A. F.; Dimitriou R.; Parvizi J.; Babis G. C. Biology of Implant Osseointegration. J. Musculoskelet. Neuronal Interact. 2009, 9, 61–71. 19516081

[ref14] Cameron H. U.; Pilliar R. M.; Macnab I. The Effect of Movement on the Bonding of Porous Metal to Bone. J. Biomed. Mater. Res. 1973, 7, 301–311. 10.1002/jbm.820070404. 4737675

[ref15] Puleo D.; Nanci A. Understanding and Controlling the Bone–Implant Interface. Biomaterials 1999, 20, 2311–2321. 10.1016/s0142-9612(99)00160-x. 10614937

[ref16] Alla R. K.; Ginjupalli K.; Upadhya N.; Shammas M.; Krishna R. Surface Roughness of Implants: A Review. Trends Biomater. Artif. Organs 2011, 25, 112.

[ref17] Murphy C. M.; Haugh M. G.; O’Brien F. J. The Effect of Mean Pore Size on Cell Attachment, Proliferation and Migration in Collagen–Glycosaminoglycan Scaffolds for Bone Tissue Engineering. Biomaterials 2010, 31, 461–466. 10.1016/j.biomaterials.2009.09.063. 19819008

[ref18] Tranquillo R. T. Self-Organization of Tissue-Equivalents: The Nature and Role of Contact Guidance. Biochemical Society Symposium, 1999; Vol. 65, pp 27–42. 10320931

[ref19] Colombo P.; Mera G.; Riedel R.; Soraru G. D. Polymer-derived Ceramics: 40 Years of Research and Innovation in Advanced Ceramics. J. Am. Ceram. Soc. 2010, 93, 1805–1837. 10.1111/j.1551-2916.2010.03876.x.

[ref20] Carlomagno C.; Speranza G.; Aswath P.; Sorarù G. D.; Migliaresi C.; Maniglio D. Breath figures decorated silica-based ceramic surfaces with tunable geometry from UV cross-linkable polysiloxane precursor. J. Eur. Ceram. Soc. 2018, 38, 1320–1326. 10.1016/j.jeurceramsoc.2017.10.005.

[ref21] Carlomagno C.; Motta A.; Sorarù G.; Aswath P.; Migliaresi C.; Maniglio D. Breath Figures Decorated Silicon Oxinitride Ceramic Surfaces with Controlled Si Ions Release for Enhanced Osteoinduction. J. Biomed. Mater. Res. 2018, 107, 1284 10.1002/jbm.b.34221. 30318728

[ref22] Carlisle E. M. Silicon: A Requirement in Bone Formation Independent of Vitamin D 1. Calcif. Tissue Int. 1981, 33, 27–34. 10.1007/bf02409409. 6257332

[ref23] Gomez-Vega J. M.; Saiz E.; Tomsia A. P.; Marshall G. W.; Marshall S. J. Bioactive Glass Coatings with Hydroxyapatite and Bioglass® Particles on Ti-Based Implants. 1. Processing. Biomaterials 2000, 21, 105–111. 10.1016/s0142-9612(99)00131-3. 10632392

[ref24] Sun W.; Ji J.; Shen J. Rings of Nanoparticle-Decorated Honeycomb-Structured Polymeric Film: The Combination of Pickering Emulsions and Capillary Flow in the Breath Figures Method. Langmuir 2008, 24, 11338–11341. 10.1021/la8024217. 18800817

[ref25] Sun W.; Shao Z.; Ji J. Particle-Assisted Fabrication of Honeycomb-Structured Hybrid Films via Breath Figures Method. Polymer 2010, 51, 4169–4175. 10.1016/j.polymer.2010.07.008.

[ref26] Albella J. M.; Jiménez Guerrero I.; Gómez-Aleixandre C.; Albertdi A. Bol. Soc. Esp. Ceram. Vidrio 2007, 46 (4), 171–176. 10.3989/cyv.2007.v46.i4.233.

[ref27] Padaki M.; Isloor A. M.; Nagaraja K. K.; Nagaraja H. S.; Pattabi M. Conversion of Micro Fi Ltration Membrane into Nano Fi Ltration Membrane by Vapour Phase Deposition of Aluminium for Desalination Application. Desalination 2011, 274, 177–181. 10.1016/j.desal.2011.02.007.

[ref28] Lanzarini-lopes M.; Cruz B.; Garcia-segura S.; Alum A.; Abbaszadegan M.; Westerho P. Nanoparticle and Transparent Polymer Coatings Enable UV - C Side- Emission Optical Fibers for Inactivation of Escherichia Coli in Water. Environ. Sci. Technol. 2019, 53, 18 10.1021/acs.est.9b01958. 31397559

[ref29] Wales D. J.; Kitchen J. A. Surface - Based Molecular Self - Assembly: Langmuir - Blodgett Films of Amphiphilic Ln (III) Complexes. Chem. Cent. J. 2016, 10, 72 10.1186/s13065-016-0224-6. 27994637 PMC5125037

[ref30] Yang Y.; Fang Z.; Chen X.; Zhang W.; Xie Y.; Chen Y.; Liu Z.; Yuan W. An Overview of Pickering Emulsions: Solid-Particle Materials, Classification, Morphology, and Applications. Front. Pharmacol. 2017, 8, 287 10.3389/fphar.2017.00287. 28588490 PMC5440583

[ref31] Yang P.; Huang J.; Sun W.; Wei Y.; Liu Y.; Ding L.; Bao J.; Chen Z.-R. Exploration of Selective Decoration of Janus Silica Particles within Polymeric Patterned Pore Arrays. RSC Adv. 2016, 6, 55860–55866. 10.1039/c6ra10035j.

[ref32] Ma H.; Fan D.; Li G.; Xia X.; Guo H.; Du B.; Wei Q. Honeycomb-Structured Porous Films Prepared from Polymer Nanocomposites of Gold Nanorods. J. Inorg. Organomet. Polym. Mater. 2013, 23, 587–591. 10.1007/s10904-012-9817-2.

[ref33] Li H.; Jia Y.; Du M.; Fei J.; Zhao J.; Cui Y.; Li J. Self-Organization of Honeycomb-like Porous TiO 2 Films by Means of the Breath-Figure Method for Surface Modification of Titanium Implants. Chem.—Eur. J. 2013, 19, 5306 10.1002/chem.201203353. 23447368

[ref34] Wu X. H.; Wu Z. Y.; Su J. C.; Yan Y. G.; Yu B. Q.; Wei J.; Zhao L. M. Nano-Hydroxyapatite Promotes Self-Assembly of Honeycomb Pores in Poly (L-Lactide) Films through Breath-Figure Method and MC3T3-E1 Cell Functions. RSC Adv. 2015, 5, 6607–6616. 10.1039/c4ra13843k.

[ref35] Jeong J.; Kim J. H.; Shim J. H.; Hwang N. S.; Heo C. Y. Bioactive Calcium Phosphate Materials and Applications in Bone Regeneration. Biomater. Res. 2019, 23, 4 10.1186/s40824-018-0149-3. 30675377 PMC6332599

[ref36] Cai Y.; Liu Y.; Yan W.; Hu Q.; Tao J.; Zhang M.; Shi Z.; Tang R. Role of Hydroxyapatite Nanoparticle Size in Bone Cell Proliferation. J. Mater. Chem. 2007, 17, 3780–3787. 10.1039/b705129h.

[ref37] Halldin A.; Ander M.; Jacobsson M.; Hansson S. Simulation of the Mechanical Interlocking Capacity of a Rough Bone Implant Surface during Healing. Biomed. Eng. Online 2015, 14, 45 10.1186/s12938-015-0038-0. 25994839 PMC4440247

[ref38] Ke B.-B.; Wan L.-S.; Li Y.; Xu M.-Y.; Xu Z.-K. Selective Layer-by-Layer Self-Assembly on Patterned Porous Films Modulated by Cassie–Wenzel Transition. Phys. Chem. Chem. Phys. 2011, 13, 4881–4887. 10.1039/c0cp01229g. 21221432

[ref39] Le T. C.; Zhai J.; Chiu W.-H.; Tran P. A.; Tran N. Janus Particles: Recent Advances in the Biomedical Applications. Int. J. Nanomedicine 2019, 14, 6749 10.2147/ijn.s169030. 31692550 PMC6711559

[ref40] Jiang S.; Chen Q.; Tripathy M.; Luijten E.; Schweizer K. S.; Granick S. Janus Particle Synthesis and Assembly. Adv. Mater. 2010, 22, 1060 10.1002/adma.200904094. 20401930

[ref41] Walther A.; Müller A. H. E. Janus Particles. Soft Matter 2008, 4, 663–668. 10.1039/b718131k. 32907169

[ref42] Walther A.; Müller A. H. E. Janus Particles: Synthesis, Self-Assembly, Physical Properties, and Applications. Chem. Rev. 2013, 113, 5194–5261. 10.1021/cr300089t. 23557169

[ref43] Maniglio D.; Ding Y.; Wang L.; Migliaresi C. One-Step Process to Create Porous Structures in Cross-Linked Polymer Films via Breath-Figure Formations during in Situ Cross-Linking Reactions. Polymer 2011, 52, 5102–5106. 10.1016/j.polymer.2011.08.054.

[ref44] Chen M.; Tan J.; Lian Y.; Liu D. Preparation of Gelatin Coated Hydroxyapatite Nanorods and the Stability of Its Aqueous Colloidal. Appl. Surf. Sci. 2008, 254, 2730–2735. 10.1016/j.apsusc.2007.10.011.

[ref45] Speranza G.; Canteri R. RxpsG a New Open Project for Photoelectron and Electron Spectroscopy Data Processing. SoftwareX 2019, 10, 100282 10.1016/j.softx.2019.100282.

[ref46] Gentili D.; Foschi G.; Valle F.; Cavallini M.; Biscarini F. Applications of Dewetting in Micro and Nanotechnology. Chem. Soc. Rev. 2012, 41, 4430 10.1039/c2cs35040h. 22491348

[ref47] Xue L.; Han Y. Inhibition of dewetting of thin polymer films. Prog. Mater. Sci. 2012, 57, 947–979. 10.1016/j.pmatsci.2012.01.003.

[ref48] Hughes J. M.; Cameron M.; Crowley K. D. Structural Variations in Natural F, OH, and Cl Apatites. Am. Mineral. 1989, 74, 870–876.

[ref49] Yi J.; Jeong H.; Park J. Modulation of Nanoparticle Separation by Initial Contact Angle in Coffee Ring Effect. Micro Nano Syst. Lett. 2018, 6, 17 10.1186/s40486-018-0079-9.

[ref50] Alvi M. A. U. R.; Khalid M. W.; Ahmad N. M.; Niazi M. B. K.; Anwar M. N.; Batool M.; Cheema W.; Rafiq S. Polymer Concentration and Solvent Variation Correlation with the Morphology and Water Filtration Analysis of Polyether Sulfone Microfiltration Membrane. Adv. Polym. Technol. 2019, 2019, 1 10.1155/2019/8074626.

[ref51] Song W.-H.; Jun Y.-K.; Han Y.; Hong S.-H. Biomimetic Apatite Coatings on Micro-Arc Oxidized Titania. Biomaterials 2004, 25, 3341–3349. 10.1016/j.biomaterials.2003.09.103. 15020106

[ref52] Durdu S.; Deniz Ö. F.; Kutbay I.; Usta M. Characterization and Formation of Hydroxyapatite on Ti6Al4V Coated by Plasma Electrolytic Oxidation. J. Alloys Compd. 2013, 551, 422–429. 10.1016/j.jallcom.2012.11.024.

[ref53] Casaletto M. P.; Kaciulis S.; Mattogno G.; Mezzi A.; Ambrosio L.; Branda F. XPS Characterization of Biocompatible Hydroxyapatite–Polymer Coatings. Surf. Interface Anal. 2002, 34, 45–49. 10.1002/sia.1249.

[ref54] He L.; Dong G.; Deng C. Effects of Strontium Substitution on the Phase Transformation and Crystal Structure of Calcium Phosphate Derived by Chemical Precipitation. Ceram. Int. 2016, 42, 11918–11923. 10.1016/j.ceramint.2016.04.116.

[ref55] Bhattacharjee B. N.; Mishra V. K.; Rai S. B.; Parkash O.; Kumar D. Structure of Apatite Nanoparticles Derived from Marine Animal (Crab) Shells: An Environment-Friendly and Cost-Effective Novel Approach to Recycle Seafood Waste. ACS Omega 2019, 4, 12753–12758. 10.1021/acsomega.9b00134. 31460398 PMC6681996

[ref56] Pecheva E.; Pramatarova L.; Toth A.; Hikov T.; Fingarova D.; Stavrev S.; Iacob E.; Vanzetti L. Effect of Nanodiamond Particles Incorporation in Hydroxyapatite Coatings. ECS Trans. 2009, 25, 403–410. 10.1149/1.3204431.

[ref57] Bouvet D.; Clivaz P. A.; Dutoit M.; Coluzza C.; Almeida J.; Margaritondo G.; Pio F. Influence of Nitrogen Profile on Electrical Characteristics of Furnace-or Rapid Thermally Nitrided Silicon Dioxide Films. J. Appl. Phys. 1996, 79, 7114–7122. 10.1063/1.361481.

[ref58] Shallenberger J. R.; Cole D. A.; Novak S. W. Characterization of Silicon Oxynitride Thin Films by X-Ray Photoelectron Spectroscopy. J. Vac. Sci. Technol., A 1999, 17, 1086–1090. 10.1116/1.582038.

[ref59] Yim C. J.; Ko D.-H.; Park S. H.; Lee W. J.; Cho M.-H. Effect of Incorporated Nitrogen on the Band Alignment of Ultrathin Silicon-Oxynitride Films as a Function of the Plasma Nitridation Conditions. J. Korean Phys. Soc. 2011, 58, 1169–1173. 10.3938/jkps.58.1169.

[ref60] Dong H.; Chen K.; Wang D.; Li W.; Ma Z.; Xu J.; Huang X. A New Luminescent Defect State in Low Temperature Grown Amorphous SiN XOy Thin Films. Phys. Status Solidi C 2010, 7, 828–831. 10.1002/pssc.200982770.

