# Supplementary material for: Ultrasound-Assisted Hydroxyapatite-Decorated Breath-Figure Polymer-Derived Ceramic Coatings for Ti6Al4V Substrates
Source: ACS Appl Mater Interfaces. 2020 Oct 27;12(45):50772–83. doi: 10.1021/acsami.0c08849 (PMC8016169; doi:10.1021/acsami.0c08849)
Supplement: Supplementary file 1 — am0c08849_si_001.pdf [file am0c08849_si_001.pdf]

Supporting Information

Ultrasound assisted Hydroxyapatite decorated Breath Figure polymer derived ceramic coatings for Ti6Al4V substrates

Simone Murchio[# +], *Yifu Ding* [§], *Giorgio Speranza* [& †], *Gian Domenico Sorarù* [#], *Devid Maniglio* [# +,*]

# Department of Industrial Engineering, University of Trento, via Sommarive 9, Povo, 38123, Trento, Italy.

+ BIOTech Research Center, University of Trento, via delle Regole 101, Trento, Italy and European Institute of Excellence on Tissue Engineering and Regenerative Medicine, Trento, Italy.

§ Department of Mechanical Engineering, 427 UCB University of Colorado, Boulder, CO 80309-0427, USA.

& Fondazione Bruno Kessler, via Sommarive 18, Povo, 38123, Trento, Italy.

† Institute of Photonics and Nanotechnologies – CNR, via alla Cascata 56/C Povo, 38123 Trento, Italy.

**Corresponding author*: devid.maniglio@unitn.it, Tel: (+39) 0461 282751

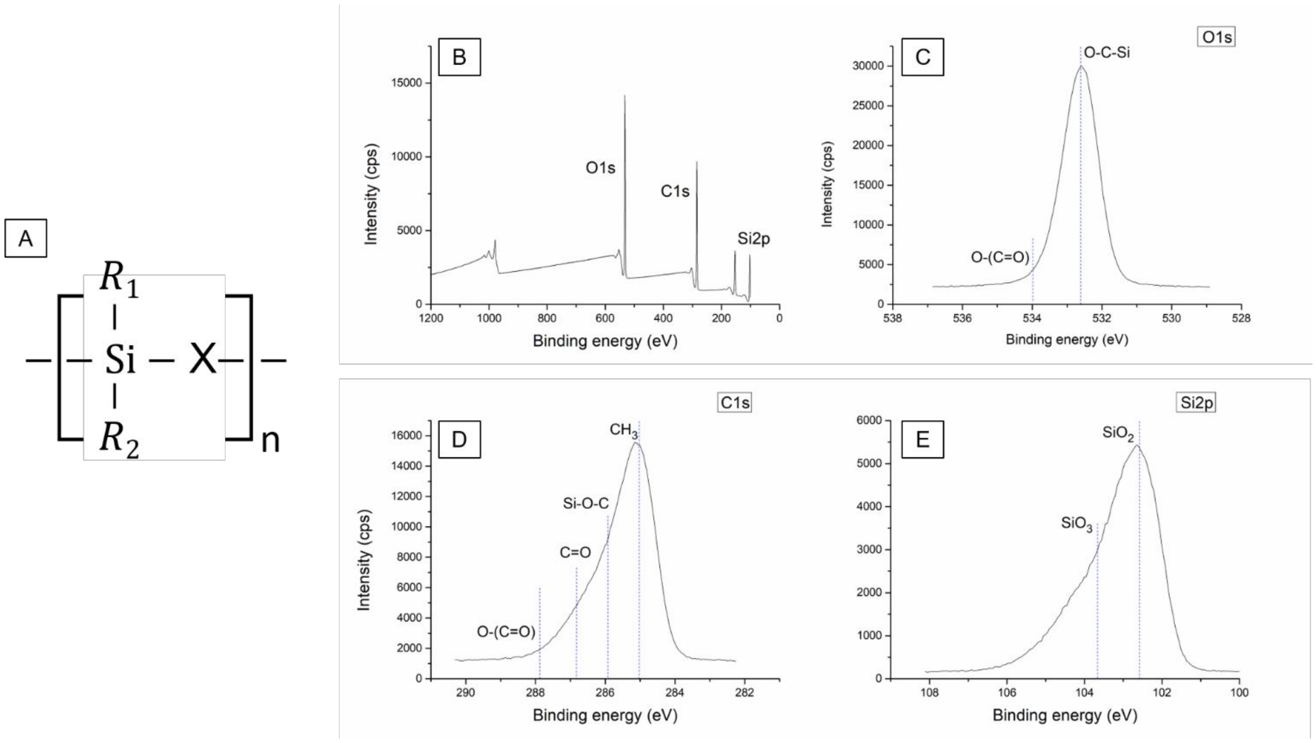

Figure S1 - A) Generic chemical structure of alkoxy silicone (Loctite® 5248™). B) XPS survey spectra of Loctite and the deconvolution of the main peaks: namely, O1s (C), C1s (D) and Si2p (E). The XPS analysis on pure Loctite was promoted to evaluate the Si:O:C ratio of the material.

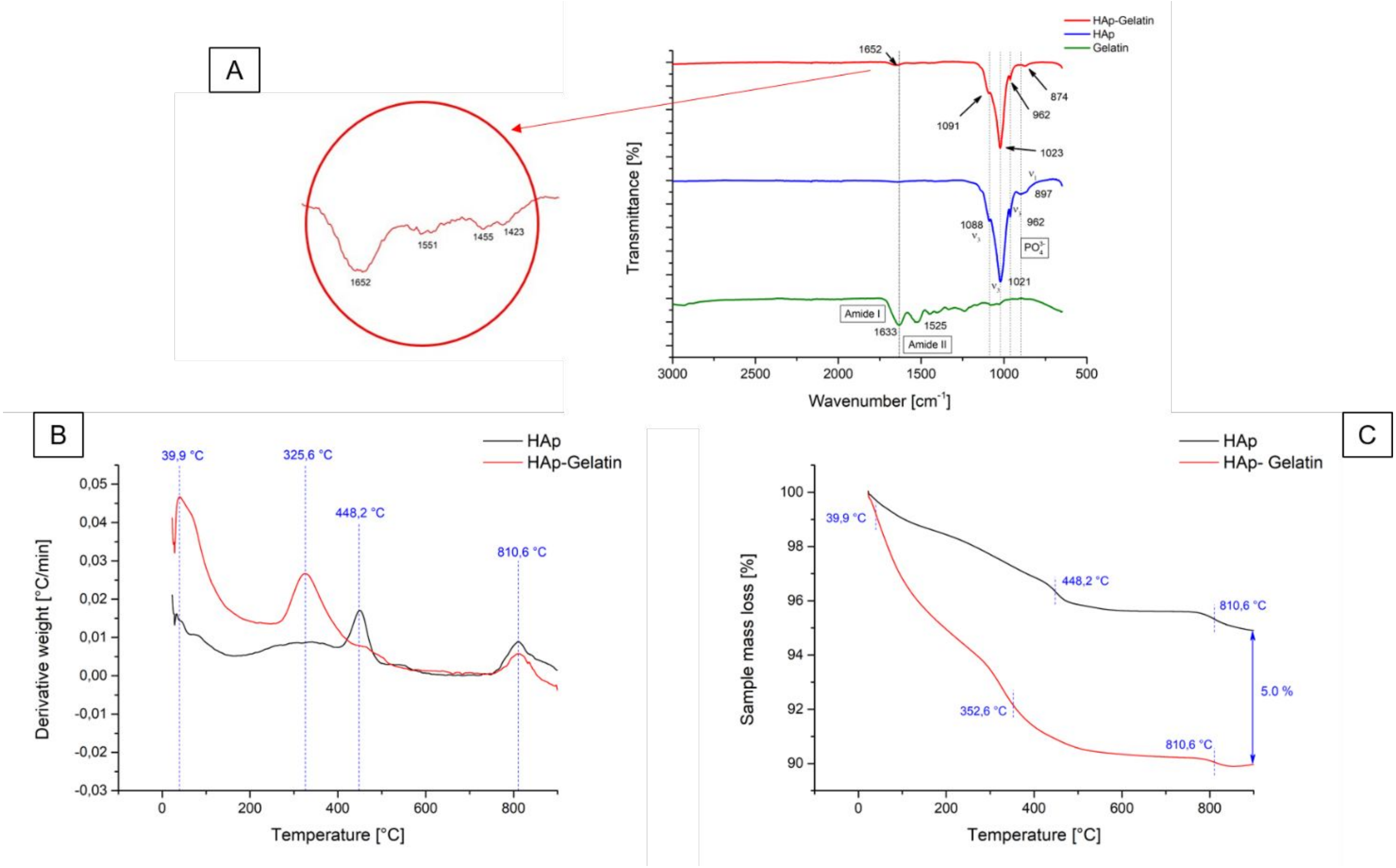

Figure S2– A) FTIR spectra comparison among synthesized gelatin-coated HAp, commercial HAp nanopowder and commercial gelatin is reported. As visible gelatin-coated HAp NPs reveal the presence of Amide I and Amide II bands at 1652and 1551 cm^{-1}, thus confirming the gelatin deposition onto the HAp NPs. Further confirmations of the gelatin coating are given by DTA and TGA spectra (Fig. 5B and Fig. 5C). A weight loss mass difference of 5% has been detected between the synthesized NPs and the commercial HAp nanopowder. This difference is related to the gelatin burn out at about 350°C.

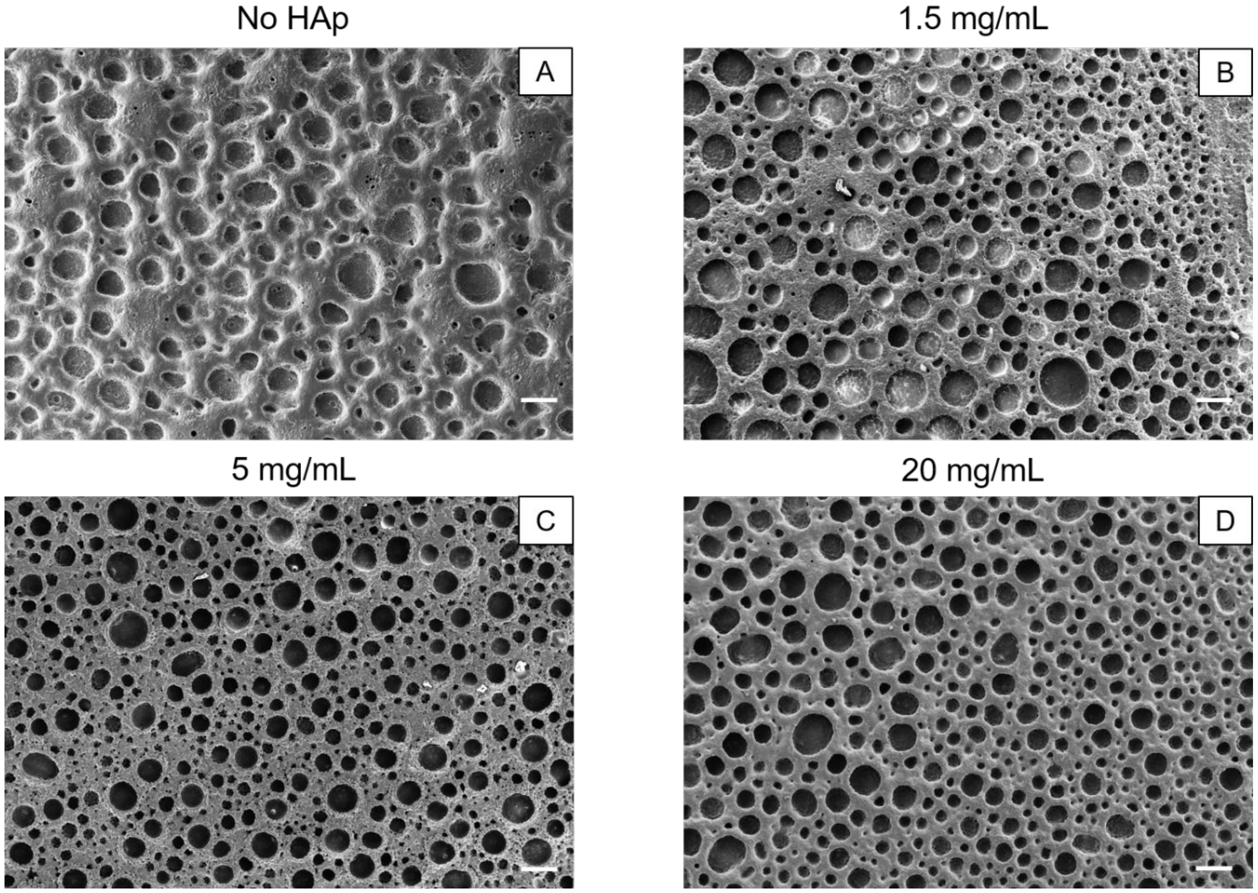

Figure S3–FE-SEM images of the four samples tested for the circularity and statistical analyses of the Pickering emulsion effect. From a visual inspection, the sample without HAp NPs shows a less regular structure, particularly considering the pore morphology. Pores are more elliptical and less regular with respect to the sample at 20 mg/mL (Fig. 2D).

Table S1 – Pore areas and circularity test statistical values are reported for 4 different tests at an increasing HAp NP concentration. For both statistical analysis, Q1, median, Q3 and interquartile range (IQR) are given.

**Test**	**Pore size**								**Circularity**			
	**Q1**		**Median**		**Q3**		**IQR**					
	**Area [μm²]**	**ϕ_{eq} [μm]**	**Area [μm²]**	**ϕ_{eq} [μm]**	**Area [μm²]**	**ϕ_{eq} [μm]**	**Area [μm²]**	**ϕ_{eq} [μm]**	**Q1**	**Median**	**Q3**	**IQR**
**No HAp**	1894	49	2727	59	3924	71	2030	22	0.71	0.75	0.79	0.08
**1.5 mg/mL**	1022	36	1748	47	2989	62	1967	26	0.65	0.76	0.82	0.17
**5 mg/mL**	901	34	1434	43	2284	54	1384	20	0.71	0.80	0.86	0.15
**20 mg/mL**	891	34	1404	42	2212	53	1318	19	0.80	0.84	0.87	0.07
